# Complete mitochondrial genome of *Macromia manchurica* Asahina, 1964 (Odonata: Macromiidae)

**DOI:** 10.1080/23802359.2022.2157197

**Published:** 2023-01-01

**Authors:** Chae-Hui An, Kyeong-Sik Cheon, Ji-Eun Jang, Hwang-Goo Lee

**Affiliations:** Department of Biological Science, Sangji University, Wonju, South Korea

**Keywords:** *Macromia manchurica*, complete mitochondrial genome, phylogeny

## Abstract

We describe the first time sequencing and assembly of the complete mitochondrial genome of *Macromia manchurica* Asahina, 1964 (Odonata; Macromiidae; Macromia). The mitochondrial genome of *M. manchurica* was found to be 15,560 bp. It contains thirteen protein-coding genes (PCGs), 22 transfer RNAs (tRNAs), two ribosomal RNAs (rRNAs), and AT-rich region. The overall base composition of *A. japonicus* is A-38.6%, C-17.0%, G-12.5%, and T-31.9%. A phylogenetic analysis of 14 species within the order Odonata and order Ephemeroptera suggested that *Macromia amphigena* is most closely related to *M. manchurica*.

## Introduction

**Figure 1.**
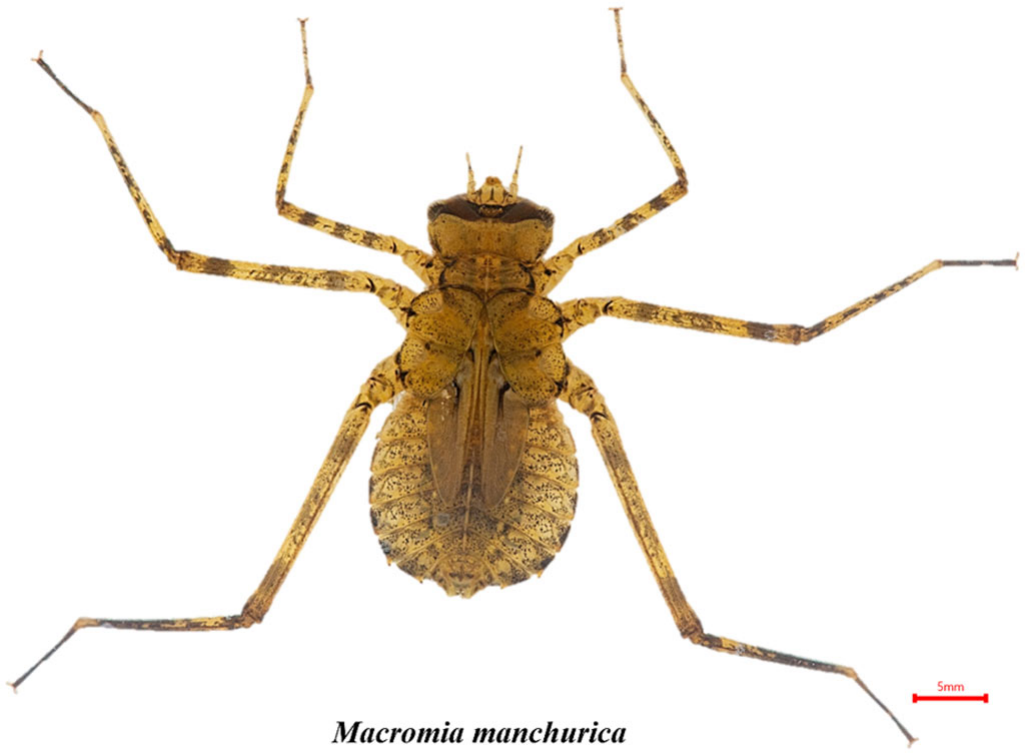
*Macromia manchurica* Asahina, 1964 (Odonata; Macromiidae; Macromia) larva image. Unlike *M. amphigena* larva, there are no setae on the legs.

Macromiidae belongs to the Odonata and consists of 125 species (Dijkstra et al. 2013; Greiner et al., 2019). *Macromia manchurica* (Odonata, Macromiidae) had been classified as a genus of *Macromia* (Lieftinck 1955; Figure 1). This species is widely distributed in Korea, Japan, China and Russia. In the genus *macromia* distributed in Korea, it is known as *Macromia amphigena*, *M. manchurica*, *Macromia daimoji*. Currently, only the mitochondrial complete genome sequence of *M. amphigena* and *M. daimoji* have been reported (Kim et al. 2018; An et al. 2022). Also we report the complete mt genome of *M. manchurica* to provide genetic information that can be used for various studies in the future.

**Figure 2.**
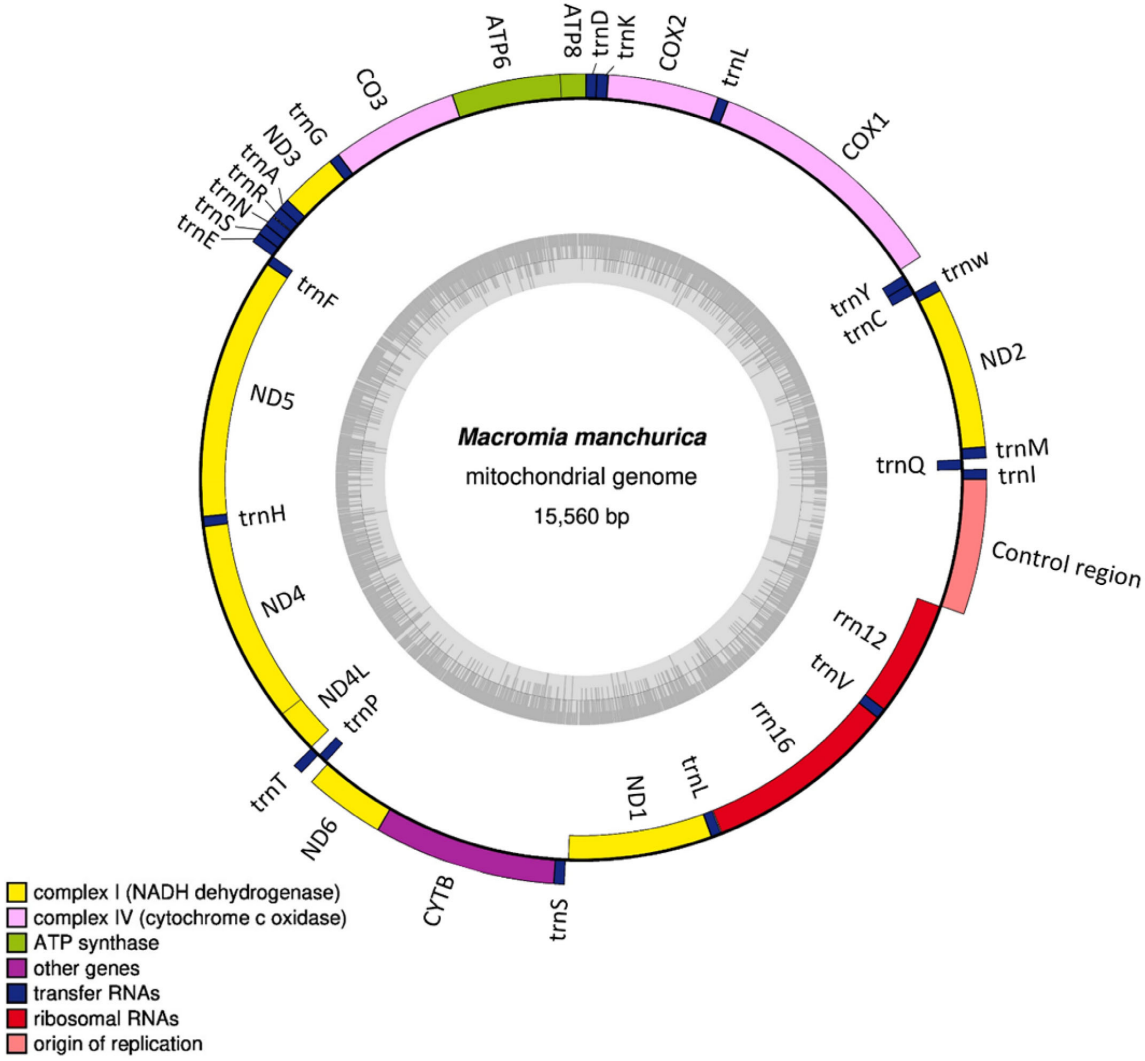
Mitochondrial genome map of the *Macromia manchurica.* A circular mitochondrial genome map was drawn using OGDRAW program (Greiner et al. 2019).

## Materials and methods

**Figure 3.**
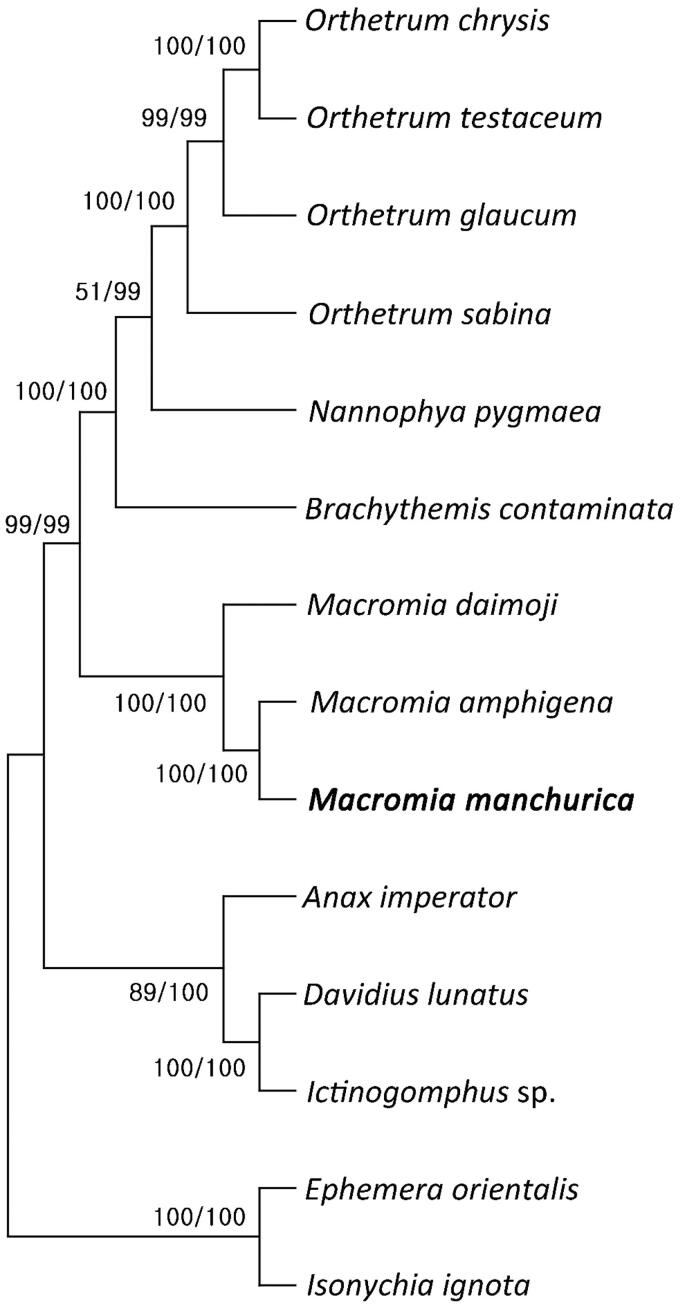
Phylogenetic tree of 12 order Odonata including *M. manchurica* and two outgroup species in order Ephemeroptera. Reconstruction of maximum likelihood (ML) and neighbor joining (NJ) trees was based on 15 genes (13 PCGs, two rRNA). Numbers at the branches represent the bootstrap support values for ML (left) and NJ (right), respectively. Branching patterns and branch lengths follow the results of ML analysis. The following sequences were used: *O. Chrysis* KU361233 (Yong et al. 2016), *O. testaceum* KU361235 (Yong et al. 2016), *O. glaucum* KU361232 (Yong et al. 2016), *O. sabina* KU361234 (Yong et al. 2016), *N. pygmaea* KY402222 (Jeong et al. 2017), *B. contaminata* KM658172 (Yu et al. 2016), *M. daimoji* NC_041425 (Kim et al. 2018), *M. amphigena* MZ504971 (An et al. 2022), *A. imperator* KX161841 (Herzog et al. 2016), *D. lunatus* EU591677 (Lee et al. 2009), *Ictinogomphus* sp. KM244673 (Tang et al. 2014), *E. orientalis* EU591678 (Lee et al. 2009), *I. ignota* HM143892 (unpublished).

*Macromia manchurica* specimen (voucher number: SJAEMM001; HG Lee, morningdew@sangji.ac.kr) used in mitochondrial genome analysis was collected from Yeongokcheon, Yeongok-myeon, Gangneung-si, Gangwon-do (37°50′47.81″N, 128°48′26.01″E). The collected specimen were stored in the Department of Biological Science of Sangji University in Korea. Ethics approval for this study was obtained from the institutional Ethics Committee of Sangji University. We did not collect material from any privately owned or protected area that required permission. Total genomic DNA was extracted from the specimen using a DNeasy Blood and Tissue Kit (Qiagen, Hilden, Germany). Genome sequencing was performed on MiSeq (Illumina Inc., San Diego, CA) platform. The treatments of raw data were performed by using Geneious prime 2022.2.1 (Biomatters Ltd, Auckland, New Zealand). To reveal the phylogenetic position of *M. manchurica*, 13 species downloaded and their GenBank accession numbers are provided in Figure 3. We also compared each gene to the previously published mitochondrial genome of *M. amphigena* (MZ504971), which was suggested to be the most closely related species (An et al. 2022). *Isonychia ignota* and *Ephemera orientalis* were used as the outgroups. The sequences were aligned using MAFFT (Katoh et al. 2002) and the maximum likelihood (ML) and neighbor joining (NJ) trees were made by MEGA X (Kumar et al. 2018).

## Results

The assembled mitogenome of *M. manchurica* (GenBank accession No: MZ504972) showed a length of 15,560 bp. Also, the total GC content is 16.8% and the total nucleotide composition is A − 38.6%, C − 17.0%, G − 12.5%, and T − 31.9%. The mitogenome consists of thirteen protein-coding genes (PCGs), 22 tRNA genes, and two ribosomal RNA (rRNA) genes (Figure 2). The genome structure, gene order, and total gene number of *M. manchurica* mt genome is identically same to *M. amphigena* (MZ504971), which is closely related genus. The two independent phylogenetic trees yielded the same topology. *Macromia manchurica* formed a monophyly with *M. amphigena* with a high support value (BS = 100) (Figure 1).

## Conclusion

The mitochondrial genome of *M. manchurica* was 15,560 bp and contained thirteen protein-coding genes, 22 transfer RNAs, two ribosomal RNAs and AT-rich region. A phylogenetic analysis of fourteen complete mitochondrial genomes of the registered Order Odonata and Order Ephemeroptera suggested that *M. amphigena* was most closely related to *M. manchurica*. Also, among the genus *macromia* inhabiting Korea, the *M. manchurica* was more closely related to the *M. amphigena* than the *M. daimoji*. We expect that the present results will facilitate further investigations into the phylogenetic relationship of the genus *Macromia*.

## Data Availability

The genome sequence data that support the findings of this study are openly available in GenBank of NCBI at https://www.ncbi.nlm.nih.gov. under the accession no. MZ504972. The associated BioProject, SRA, and Bio-Sample numbers are PRJNA744014, SRR15092394 and SAMN20065584, respectively.
